# Primary bladder adenocarcinoma versus metastatic colorectal adenocarcinoma: a persisting diagnostic challenge

**DOI:** 10.1186/1746-1596-7-151

**Published:** 2012-11-02

**Authors:** Somak Roy, Matthew A Smith, Kathy M Cieply, Marie B Acquafondata, Anil V Parwani

**Affiliations:** 1 Department of Pathology, University of Pittsburgh Medical Center, Pittsburgh, PA, 15232, USA; 2 Florescence in-situ hybridization research and development laboratory, University of Pittsburgh Medical Center, Pittsburgh, PA, 15232, USA; 3 Division of Pathology Informatics, Department of Pathology, 5230 Centre Avenue, Room WG 07, Pittsburgh, PA, 15232, USA

**Keywords:** Bladder, Adenocarcinoma, Primary, Metastatic, Colorectal, Beta-catenin, E-cadherin

## Abstract

**Aim:**

This study attempted to distinguish primary bladder adenocarcinoma (PBA) from metastatic colonic adenocarcinomas (MCA), which is a difficult diagnostic and clinical problem.

**Methods:**

Twenty-four cases of bladder adenocarcinomas (12 primary & 12 metastatic colorectal) were included in the study with urothelial carcinoma (UC) and colonic adenocarcinoma (CA) as controls. A panel of immunohistochemical (IHC) stains along with fluorescence in-situ hybridization (FISH), using the UroVysion probe set, was performed.

**Results:**

The majority of the PBAs presented with advanced disease. Enteric histologic subtype was the most common morphological variant. Strong nuclear with cytoplasmic-membranous staining of β-catenin was seen in 75% of MCA and only 16.7% PBA (<10% staining cells). Although abnormal nuclear staining with E-cadherin was seen in both PBA and MCA, it was more frequent in former. CK-7, CK-20, villin and CDX-2 stains were not helpful in distinguishing the two entities. FISH did not reveal any unique differences in chromosomal abnormality between the two groups.

**Conclusion:**

Although there was a statistically significant difference in β-catenin and E-cadherin staining between two groups, we did not find any IHC or FISH marker that was specific for PBA. Distinction between PBA and MCA remains a diagnostic problem and clinical correlation is vital before rendering a diagnosis.

**Virtual slides:**

The virtual slides for this article can be found here: http://www.diagnosticpathology.diagnomx.eu/vs/1393156268152357

## Introduction

Primary bladder adenocarcinoma (PBA), histologically comprised entirely or almost entirely of malignant glandular elements, is a rare tumor accounting for 0.5-2% of all malignant vesical tumors [1-3]. This glandular tumor, like other variants, arises through a process of divergent differentiation in urothelial carcinoma, which is extensive enough to predominate as the only histological component [1,4-6]. It is more frequent in males in their sixth decade of life, presenting with hematuria and symptoms attributable to bladder irritation [1,7]. Two-thirds of PBA arise in the bladder cavity, especially in the posterior wall and trigone, and Approximately one-third originate from urachal remnants near the dome and anterior wall of the bladder [7,8]. Based on this, it is broadly classified as non-urachal and urachal adenocarcinoma, respectively. Although both subtypes of PBA have remarkably similar histological and immunohistochemical features, urachal subtype requires specific diagnostic criteria, put forth by Sheldon et al. [9] and Mostofi et al [10]. PBAs usually have associated surface glandular metaplasia or cystitis glandularis in the surrounding urothelial lining; however this is often difficult to document on small bladder biopsies and trans-urethral resection specimens due to ulcerated and cauterized epithelium. Approximately 90% of the vesical tumors arising in extrophied bladders are adenocarcinomas [7]. Additional risk factors include Schistsoma infection, villous adenoma and cystocele.[2,11-13] PBA is an aggressive malignancy with a tendency to present at a higher stage and is associated with a worse overall survival [1,2,13].

The more common secondary bladder adenocarcinomas include hematogenous, lymphatic or direct spread of adenocarcinoma from surrounding organs, especially colo-rectum, female genital tract, prostate, and urothelial carcinoma with focal glandular differentiation. Metastatic colonic adenocarcinoma (MCA) accounts for approximately one third of secondary bladder tumors and is virtually indistinguishable from PBA based on histomorphology and ultrastructural features. Evidence of cystitis glandularis or intestinal metaplasia can be a helpful clue in establishing the diagnosis of primary bladder adenocarcinoma; however, MCAs have been reported to colonize the surface urothelium and mimic in-situ glandular lesions [3,7,14,15].

There are relatively limited numbers of studies that have looked into the role of immunohistochemistry in differentiating PBA from secondary bladder carcinoma, specifically MCA [2,3,14-17]. The role of β-catenin in differentiating PBA from MCA was first reported by Wang et al. [3] in 2001. They attempted to analyze abnormal nuclear localization of β-catenin in colonic adenocarcinomas (CA) due to dysregulation of the wnt pathway, in the setting of PBA. Currently, there is limited published data on the use of β-catenin in differentiating PBA from MCA [2,3,14,18]. In this study, we analyzed the role of a panel of six IHC stains in differentiating PBA from MCA. We also attempted to detect any difference in underlying chromosomal abnormality by FISH using UroVysion probe set.

## Materials and methods

The Institutional Review Board of University of Pittsburgh approved this study.Twenty-four cases of bladder adenocarcinomas diagnosed and treated at University of Pittsburgh Medical Center between 1999 and 2010 were identified which included 12 cases each of PBA and MCA. We also included 5 cases each of urothelial carcinoma (UC) and colonic adenocarcinoma (CA) as controls in this study. Formalin-fixed, paraffin embedded tissue blocks along with hematoxylin and eosin (H&E) stained slides were retrieved from the archives. The pathologists, in order to confirm the diagnosis and exclude urothelial carcinoma with focal glandular differentiation, reviewed the slides independently. Cystoscopic, colonoscopic and radiological findings were reviewed to confirm the origin, location and stage of the tumor.

A panel of immunohistochemical stains was performed on formalin-fixed, paraffin embedded, unstained sections from each case (Table 1). Briefly, sections were cut at 5μ and mounted on Superfrost Plus slides and dried. Sections were deparaffinized and hydrated to deionized water. Heat induced epitope retrieval using Citrate buffer (pH 6, DAKO, Carpinteria, CA) was performed followed by endogenous Peroxidase quenching using 3% Hydrogen peroxide (Fisher Scientific, Houston, TX) and blocking (CAS, Invitrogen, Carlsbad, CA) for 10 minutes. Slides were incubated with primary antibody for 30–45 minutes, secondary antibody (Mach 2 Mouse HRP, Biocare, Concord, CA) for 30 minutes and substrate chromogen (DAKO, Carpinteria, CA) for 5 minutes. Slides were washed with TBS buffer between the incubations and finally counterstained with Harris hematoxylin, dehydrated, cleared and cover slipped.

**Table 1:** Details of immunohistochemical panel

**Antibody**	**Type**	**Dilution**	**Source**
**Cytokeratin 7**	Rabbit-Monoclonal	1:100	Biocare Medical, Concord, CA
**Cytokeratin 20**	Mouse Monoclonal	1:200	Biocare Medical, Concord, CA
**CDX**-**2**	Mouse Monoclonal	1:100	Biocare Medical, Concord, CA
**Villin**	Mouse Monoclonal	1:100	Biocare Medical, Concord, CA
β-**catenin**	Mouse Monoclonal	1:200	DAKO, Carpinteria, CA
**E**-**cadherin**	Mouse Monoclonal	1:2000	BD Transduction Labs, San Jose, CA

Cytoplasmic staining with membrane accentuation (CM) was considered the normal pattern of staining with β-catenin. Nuclear (N) staining was considered abnormal. CM staining was the expected pattern for E-cadherin whereas nuclear (N) staining was abnormal. Apical brush border staining pattern was considered positive for villin. Nuclear immunoexpression was the expected pattern with CDX2 antibody and cytoplasmic staining was considered positive for CK7 and CK20 (Table 1).

A total of nine cases (5 PBA & 4 MCA) were available for Fluorescence in-situ hybridization (FISH), which was performed using the UroVysion probe set (Abbott Molecular, Inc., Des Plaines, IL). Five cases each of UC and MCA were used as controls. Briefly, formalin-fixed paraffin-embedded sections, were mounted, and serially sectioned at 5μ intervals. The area of interest was demarcated using an H&E stained slide. FISH slides were deparaffinized in xylene, dehydrated with 100% ethanol and then pretreated for 30 min in 0.2N HCl. Slides were then digested in protease solution at 37°C. The target slide and probe were co-denatured at 90 C for 12 minutes and incubated overnight at 37°C in a humidified chamber. Post-hybridization washes were performed using 2XSSC/0.3% Igepal at 72°C for 2 minutes. Slides were air-dried in the dark and counterstained with DAPI I (Abbott Molecular, Des Plaines, IL). Analysis was performed using an Applied Imaging Workstation equipped with Chroma Technology filters containing band excitors for SpectrumOrange, FITC, DAPI. Only individual and well-delineated cells were scored. Overlapping cells were excluded from the analysis. Approximately 60 cells were analyzed in the targeted region. Statistical analysis was performed using SPSS v19.0 (IBM, Armonk, NY).

## Results

### Clinicopathologic features of primary bladder adenocarcinoma

The clinicopathologic characteristics of 12 cases of PBA are summarized in Tables 2 and 3. The age at presentation ranged from 53–87 years (mean age 64.6 years) with a male predominance [4,18]. The cases did not demonstrate any associated predisposing conditions (Schistosomiasis, cystocele, bladder exstrophy). The majority of the cases of PBAs (75%) presented at a high pathologic stage (≥ pT3). Four of 12 (33.3%) PBA cases had node positive disease and one patient (8.3%) presented with lung metastasis (Table 2) .

**Table 2:** Clinical features of primary bladder adenocarcinoma

**Case No**	**Age**	**Sex**	**Location**	**Size** (**cm**)	**Procedure**	**Stage**^¥^	**Follow**-**up** (**months**)	**Outcome**	**Residual disease**
**1**	63	F	Posterior	3.6	Cystourethrectomy	T4aN2M0	33	AWD^£^	Lymph node metastasis
**2**	54	F	Posterior & Lateral	4	Radical cystectomy	T4bN2M0	16	NED^∂^	
**3**	87	M	Posterior	6	Partial cystectomy	T1N0M0	13	NED	
**4**	53	M	Anterior	3.6	Partial cystectomy	T3N0M0	4	NED	
**5**	70	M	Posterior	6.8	TURBT^‡^	T4N0M0	2	DOD^€^	
**6**	62	M	Posterior	3.5	TURBT^‡^	T2bN0M1	18	AWD	Lung and pleural metastasis
**7**	59	M	Anterior	2.9	Partial cystectomy	T3aN0M0	132	NED	
**8**	59	M	Anterior	2.9	Partial cystectomy	T3aN0M0	128	NED	
**9**	62	M	Anterior	4	Radical cystectomy	T4N0M0	36	AWD	Lung and pleural metastasis
**10**	80	M	Posterior & Anterior	8	Radical cystectomy	T3aN1M0	2	AWD	Multiple bony metastasis
**11**	56	M	Posterior	5	Radical cystoprostatectomy	T3aN2M0	4	NED	
**12**	70	M	Multifocal^†^	0.3-0.7	Radical cystoprostatectomy	T2aN0M0	2	NED	

† − Tumor located predominantly in posterior wall with other tumor nodules in anterior and lateral walls. ‡ − TURBT – Transurethral resection of bladder tumor. ∂ - NED – No evidence of disease. € - DOD – Died of disease. £ - AWD – Alive with disease. ¥ -Pathologic stage.

**Table 3:** Pathologic features of primary bladder adenocarcinoma

**Case No**	**Histological diagnosis**	**Mucin**	**SRC**	**CC**/**CG**	**Villous**
**1**	MD AdenoCa (enteric)	+	-	+	-
**2**	PD AdenoCa (enteric)	-	+	+	+
**3**	MD AdenoCa (enteric)	+	-	+	+
**4**	MD to PD AdenoCa (enteric)	+	+	-	-
**5**	MD AdenoCa (enteric)	-	-	-	-
**6**	PD AdenoCa (SRC)	+	+	+	+
**7**	MD AdenoCa (enteric & mucinous)	+	-	+	-
**8**	MD AdenoCa (enteric & mucinous)	+	-	+	-
**9**	MD AdenoCa (enteric)	-	-	+	-
**10**	PD AdenoCa (mucinous and SRC)	+	+	-	-
**11**	Mucinous AdenoCa (enteric)	+	+	+	-
**12**	MD AdenoCa (enteric)	-	-	+	-

*MD* Moderately differentiated, *PD* Poorly differentiated, *AdenoCa* Adenocarcinoma, *SRC* Signet ring cell carcinoma, *CC* Cystitis cystica, *CG* Cystitis glandularis, *Villous* Villous adenoma.

Follow-up data was available on all the patients (2 months to 11 years), which is summarized in Table 2. Seven patients (58.3%) were alive with no evidence of disease and four patients (33.3%) were alive with evidence of disease in the form of distant metastasis (metastasis to lung and bone). Of the patients with progressive disease, case numbers 1, 9 and 10 developed metastatic disease at 33, 36 and 2 months from the time of diagnosis, respectively (Table 2).

**Figure 1:**
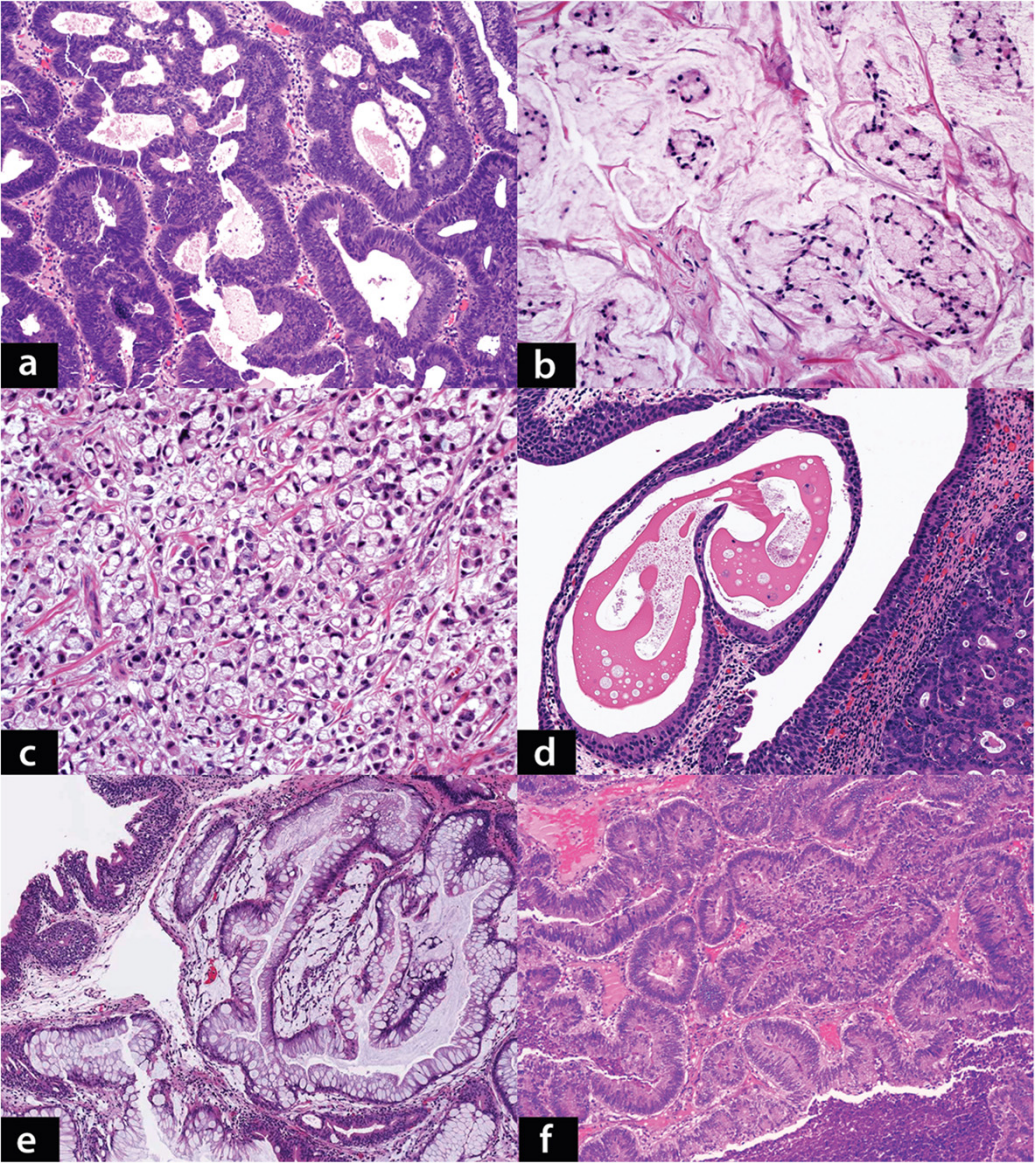
**a: Primary bladder adenocarcinoma**,** enteric type.** Moderately differentiated malignant glands are seen with dirty luminal necrosis. Elongated, enlarged and hyperchromatic nuclei with prominent stratification line the malignant glands. (Hematoxylin & eosin, x100). **b**: Primary bladder adenocarcinoma, mucinous type. Scattered small groups of tumor cells with intracytoplasmic mucin are seen in a background of abundant mucinous material (Hematoxylin & eosin, x200). **c**: Primary bladder adenocarcinoma, signet ring cell type: Tumor comprised of diffuse sheets of signet ring cells infiltrating the bladder wall (Hematoxylin & eosin, x200). **d**: Cystitis cystica involving the surface urothelium with underlying invasive primary bladder adenocarcinoma (Hematoxylin & eosin, x100). **e**: Extensive cystitis glandularis, intestinal type seen adjacent to a focus of invasive primary bladder adenocarcinoma (not seen in this image) (Hematoxylin & eosin, x40). **f**: Metastatic colorectal adenocarcinoma. Moderately differentiated, infiltrating malignant glands with morphological features similar to primary bladder adenocarcinoma see in a (Hematoxylin & eosin, x100).

Ten of the 12 (83.3%) cases of PBA were of the enteric type with varying degrees of differentiation (Figure 1a). Approximately two-third of the PBA cases originated in the posterior wall of the bladder and 1/3rd were seen to arise from the anterior wall. The average tumor size was 4.3 cm. In case number 12, there were multiple tumor nodules (0.3–0.7 cm) and although the tumor nodules were predominantly in the posterior wall, additional nodules were present in the anterior and lateral bladder walls (multifocal). Histological variations included the presence of mucin (8/12, 66.7%, Figure 1b) and signet ring cells (5/12, 41.7%, Figure 1c). Cystitis cystica and cystitis glandularis were seen in 9/12 (75%, Figure 1d, 1e) and villous adenoma was seen in 3/12 (25%) cases. All MCA cases were metastatic from the colon and rectum (Figure 1f) (Table 3).

### Immunohistochemical findings

**Figure 2:**
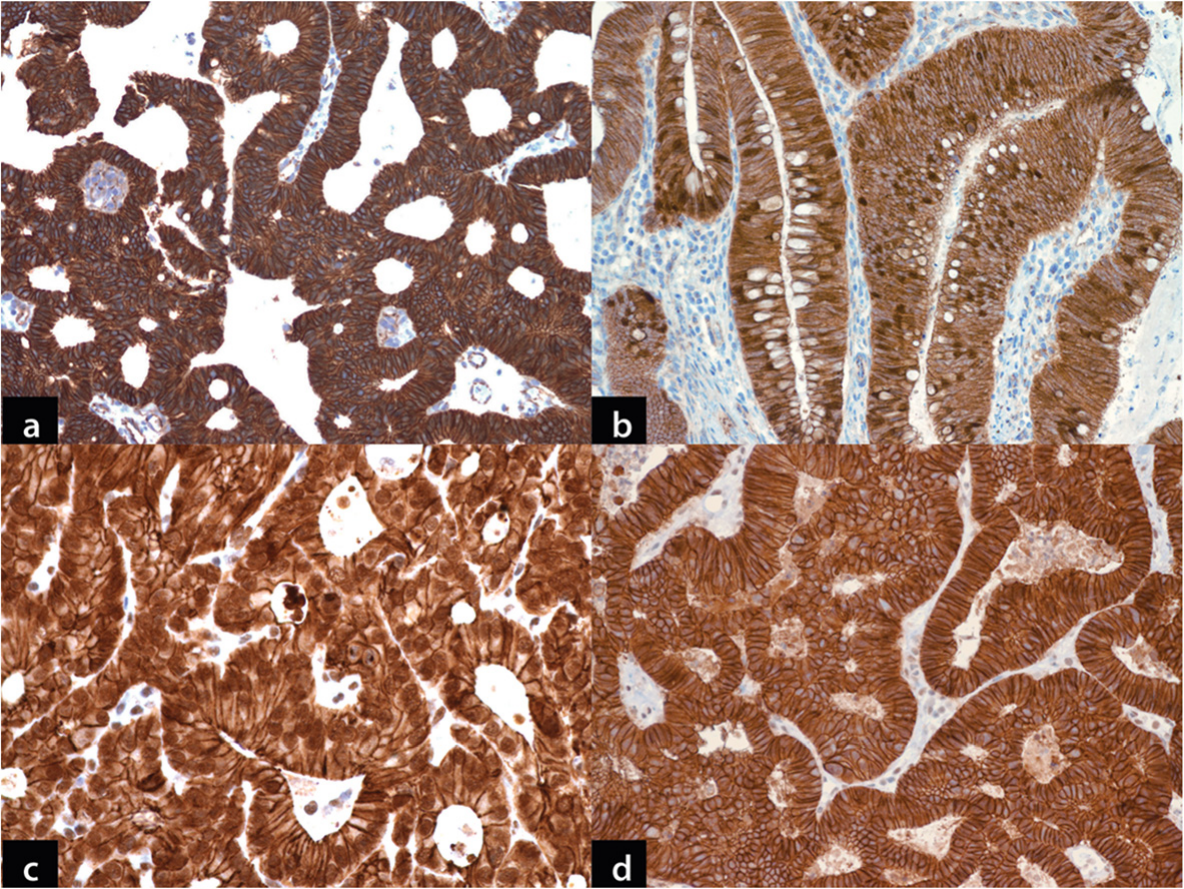
**a: Primary bladder adenocarcinoma**. Strong cytoplasmic membranous staining with β-catenin (DAB chromogen, x100). **b**: Metastatic colorectal adenocarcinoma. Strong nuclear staining in addition to cytoplasmic staining with β-catenin (DAB chromogen, x100). **c**: Primary bladder adenocarcinoma. Strong nuclear staining with e-cadherin in addition to weaker cytoplasmic staining pattern. This pattern was more frequent in this group of tumor in contrast to metastatic colorectal adenocarcinoma (DAB chromogen x200). **d**: Metastatic colorectal adenocarcinoma. Prominent cytoplasmic membranous staining with e-cadherin. Note the absence of nuclear staining (DAB chromogen, x200).

The results of immunohistochemical staining for the PBA cases are summarized in Tables 4 and 5. All PBA cases demonstrated CM staining with β-catenin stain (Figure 2a). Two cases (16.7%) demonstrated focal nuclear staining in addition (<5% cells). Nine of 12 (75%) MCAs demonstrated strong nuclear and CM staining (Figure 2b) and remaining (25%) did not demonstrate nuclear staining. The control cases of CA showed strong and diffuse nuclear as well as CM expression of β-catenin in 4/5 (80%) cases and focal (20% cells) nuclear staining in one case. Nuclear staining was not seen in UCs (Table 4).

**Table 4:** **Results of **β-**catenin and E**-**cadherin immunostaining of primary bladder adenocarcinoma**,** metastatic and primary colorectal adenocarcinoma and urothelial carcinoma**

		**PBA**	**MCA**	**CA**	**UC**
**Beta**-**Catenin**	**CM**+**N**	2 (16.7%)^*^	9 (75%)	5 (100%)	0
	**CM only**	10 (83.3%)	3 (25%)	0	5 (100%)
	**Total**	12	12	5	5
**E**-**cadherin**	**CM**+**N**	7 (58.3%)	3 (25%)	0	0
	**CM only**	5 (41.7%)	9 (75%)	5 (100%)	5 (100%)
	**Total**	12	12	5	5

^*^-Nuclear staining seen in <10% cells.

*CA* Colorectal adenocarcinoma, *CM* cytoplasmic staining with membranous accentuation, *MCA* metastatic colorectal adenocarcinoma, *N* nuclear, *UC* urothelial carcinoma.

**Table 5:** **Results of Cytokeratin 7 **&**20**,** villin and CDX**-**2 immunostaining of primary bladder adenocarcinoma**,** metastatic and primary colorectal adenocarcinoma and urothelial carcinoma**

	**PBA**	**MCA**	**CA**	**UC**
**Cytokeratin 7**	4 (33.3%)	1 (8.3%)	0	5 (100%)
**Cytokeratin 20**	12 (100%)	12 (100%)	5 (100%)	2 (40%)
**Villin**	12 (100%)	11 (91.7%)	5 (100%)	0
**CDX**-**2**	10 (83.3%)	12 (100%)	5 (100%)	0

*CA* Colorectal adenocarcinoma, *CM* cytoplasmic staining with membranous accentuation, *MCA* metastatic colorectal adenocarcinoma, *UC* urothelial carcinoma.

**Figure 3:**
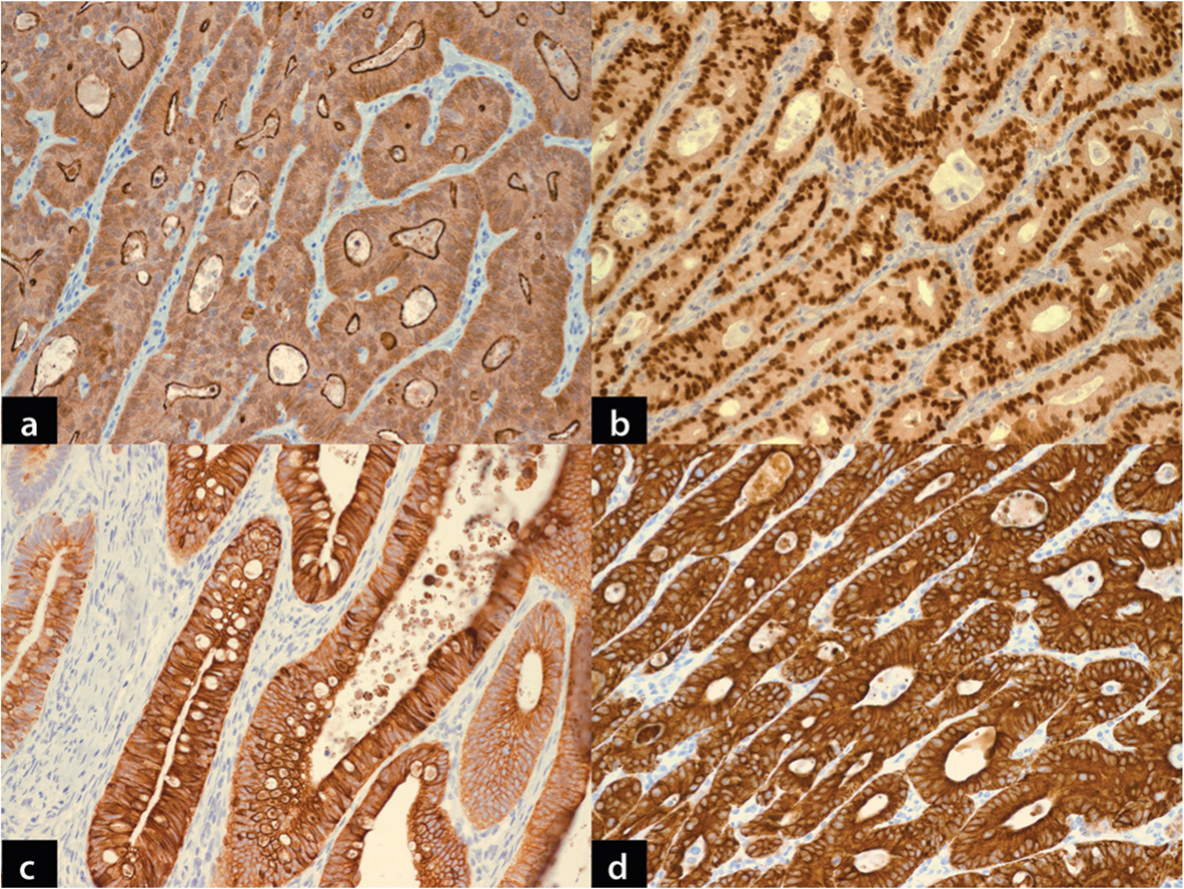
**a: Primary bladder adenocarcinoma demonstrating apical brush border staining with villin**. Very similar staining pattern was also seen in metastatic colorectal adenocarcinoma. (DAB chromogen, x100). **b**: Primary bladder adenocarcinoma demonstrating strong diffuse nuclear staining with CDX-2. Metastatic colorectal adenocarcinoma demonstrated same staining pattern. (DAB chromogen, x100). **c**: Primary bladder adenocarcinoma shows strong cytoplasmic staining with CK7. Overall, it was infrequently seen in glandular bladder tumors, however slightly more frequently in primary than metastatic adenocarcinomas. (DAB chromogen, x100). **d**: Primary bladder adenocarcinoma shows diffuse strong cytoplasmic positivity for CK20. This was also seen in majority of metastatic colorectal adenocarcinoma. (DAB chromogen, x100).

CM and nuclear staining with E-cadherin was restricted to PBA (7/12, 58.3%) and MCA (3/12, 25%) only (Figure 2c, 2d). The difference in abnormal nuclear expression of e-cadherin between PBA and MCA was not statistically significant (Fisher’s Exact test, p=0.214). However, when PBA was compared to MCA and CA combined, the difference was statistically significant (Fisher’s Exact test, p=0.046). (Table 5) Apical brush border staining with villin was seen in all cases of PBA and 11/12 (91.7%) cases of MCA (Figure 3a). CDX-2 expression was documented in 10/12 (83.3%) of PBA and all cases of MCA (Figure 3b). Four (33.3%) cases of PBA and 1 (8.3%) case of MCA demonstrated strong CK7 expression (Figure 3c). CK20 expression was seen in all cases of PBA and MCA (Figure 3d) (Table 5).

### Fluorescence in-situ hybridization results

**Figure 4:**
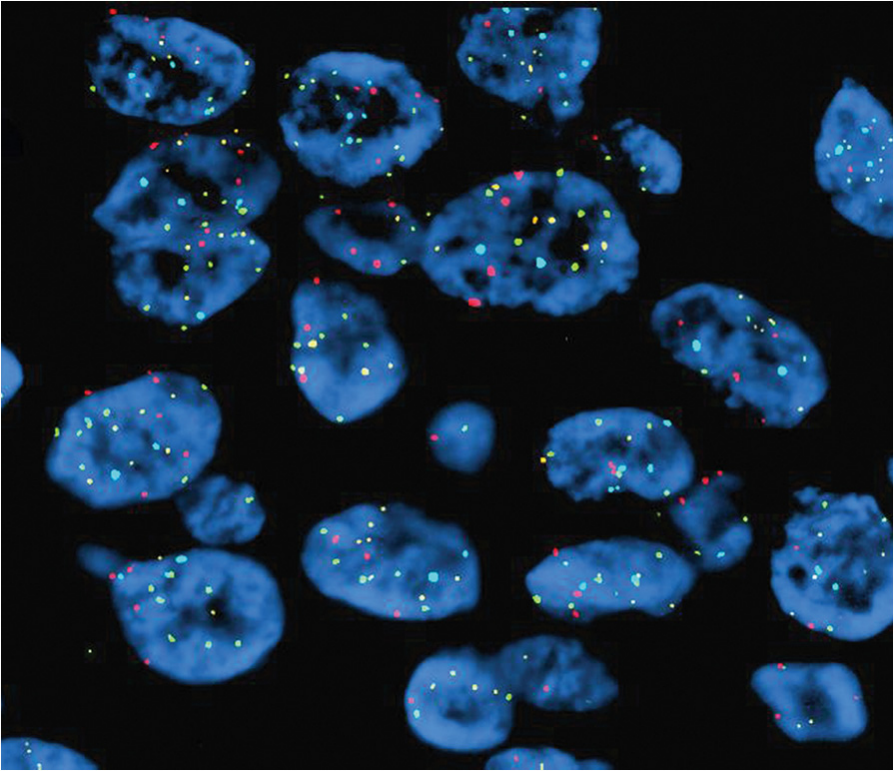
**Primary bladder adenocarcinoma.** FISH using UroVysion probe set demonstrates complete loss of yellow signal in some cells (homozygous loss 9p21) and single chromosome 3 gain in fewer cells (>2 red signals) (Red – CEP3, Green – CEP7, Aqua – CEP17 and Yellow – LSI 9p21, x600).

**Figure 5:**
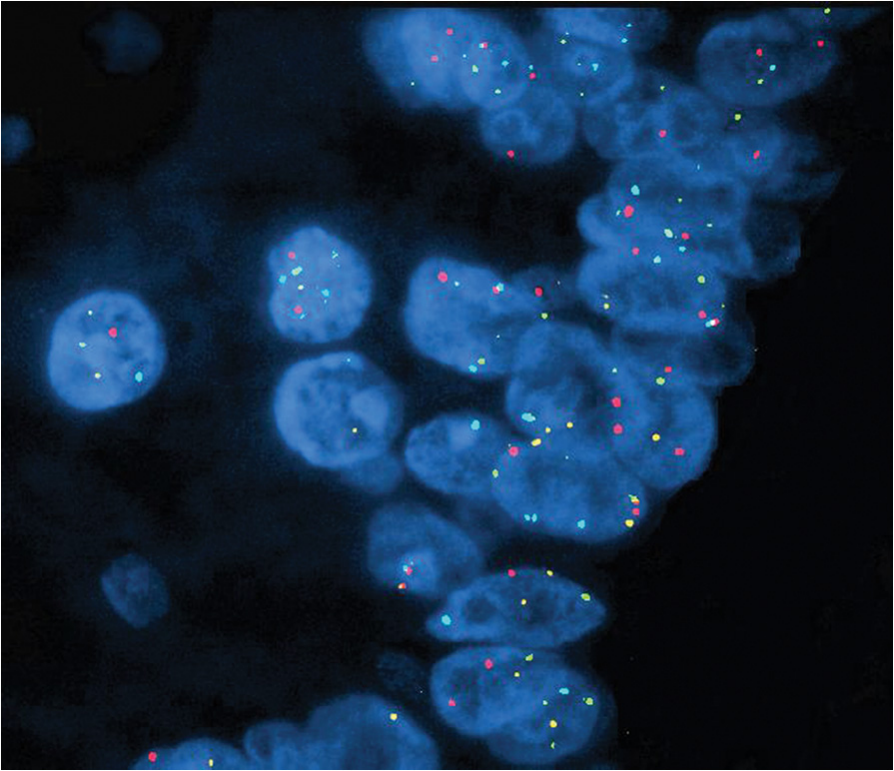
**Primary bladder adenocarcinoma.** FISH using UroVysion probe set demonstrates a polysomy pattern with more than 2 red, green and aqua signals. (Red – CEP3, Green – CEP7, Aqua – CEP17 and Yellow – LSI 9p21, x600).

The most frequent chromosomal abnormalities detected by UroVysion FISH in PBAs were 9p21 homozygous loss (HL) (6/9, 67%) (Figure 4) followed by Polysomy (PL) pattern (3/9, 33%) (Figure 5). HL, PL and single chromosome gain of chromosome 3 (SG) were seen in 3/5 (60%), 1/5 (20%), and 1/5 (20%) of PBA cases, respectively. Secondary adenocarcinoma showed HL (3/4, 75%), PL (2/4, 50%) and SG (**chromosome 7**) (1/4, 25%), respectively (Table 6).

**Table 6:** Results of FISH using UroVysion probe set

**Case #**	**FISH scores**		
	**SG**	**PL**	**HL**
**PBA**			
**Case 1**	0	0	4%
**Case 2**	0	0	12%
**Case 3**	8% (Ch 3)	0	4%
**Case 4**	0	8%	0
**Case 5**	0	0	0
**MCA**			
**Case 1**	0	84%	4%
**Case 2**	0	52%	4%
**Case 3**	0	0	8%
**Case 4**	4% (Ch 7)	0	0

*SG* Single chromosome gain; *PL* Polysomy; *HL* Homozygous loss 9p21

*PBA* Primary bladder adenocarcinoma; *MCA* Metastatic colonic adenocarcinoma *Ch* Chromosome; *FISH* fluorescence in-situ hybridization.

## Discussion

Primary bladder adenocarcinoma is a rare tumor comprising no more than 2% of all primary vesical malignancies [1-3]. in our series, the male to female ratio was 5:1 with mean age of 64.6 years. Eight of 12 cases (66.7%) originated from the posterior wall. Four tumors were seen in the anterior wall near the dome. However, they did not meet the other required criteria to be classified as urachal type adenocarcinoma. These findings were in accordance to published literature [2,3,7,8].

The average size of tumor documented in our study was 4.25 cm (range 0.7-6.8 cm). Histologically, PBA is usually well to moderately differentiated and most frequently are of the enteric type, comprised of glandular structures lined by cuboidal to columnar cells with basally located vesicular nuclei and prominent nucleoli. The cytoplasm is usually apical with or without mucin vacuoles, the former representing goblet cells found in benign and malignant colonic glands. Mitoses and dirty necrosis are frequently seen [1,3,4,7]. Varying degree of extracellular mucin may be seen with these tumors. The mucinous variant is comprised of abundant extracellular pools of mucin with floating tumor cells. Additionally, some of the tumors may also harbor signet ring cells with intracellular mucin, the latter often referred to as the signet ring cell variant of PBA [2,18].

In the current study, there was a predominance of moderately differentiated PBA (75%), all of which were of the enteric type. Extracellular mucin and signet ring cells were seen in 66.7% and 41.7% cases, respectively. Case #6 had predominance of signet ring cells (>75%) and was classified as signet ring cell adenocarcinoma. Cystitis cystica/glandularis was documented in three-quarters of the cases and a villous adenoma like lesion was seen in 25% cases. Other variants, such as hepatoid and clear cell types, were not included in this study.

Secondary bladder adenocarcinoma, which is more frequent than PBA, is represented by either direct extension of tumor from surrounding organs like the rectum, colon and female genital tract or lymphatic and hematogenous spread of tumor. MCA is by far the most frequent type in SBA. It is important to distinguish PBA from MCA for disease staging (local versus metastatic), and management [2,3,16].

Abnormal localization of β-catenin has been well documented in colonic adenocarcinomas with mutation in APC tumor suppressor gene [19]. The wild-type APC functions to degrade free cytoplasmic β-catenin, an important molecule in cadherin mediated cell-to-cell adhesion system. In patients with mutated or absent APC, β-catenin translocates to the nucleus of the cell and acts as a transactivating factor for the transcriptional factor Tcf-4 to regulate the expression of a number of downstream target genes that are believed play a role in oncogenesis [20]. Wang et al. first exploited this finding in the setting of PBA and observed absence of nuclear staining in all PBA and nuclear staining in 81% MCA. This was reported to be a helpful distinction between the two entities [3]. Subsequently, few other studies reported the utility of β-catenin staining in settings of signet ring cell adenocarcinomas of bladder and urachal adenocarcinomas [2,14,18]. In our study, nuclear staining was predominantly seen in MCA and CA unlike PBA. In two cases of PBA nuclear staining was seen in less than 5% of the tumor cells. These findings are similar to the previously reported study by Gopalan et al. [14] who reported focal nuclear staining (15% cells) in 1 of 24 cases of urachal adenocarcinomas. However, the nuclear staining was seen in the clear cell urothelial component of the tumor rather than the adenocarcinoma component itself. Overall the difference in β-catenin staining pattern between PBA and MCA was statistically significant (*χ*2 test, p=0.001).

The e-cadherin molecule forms an important component of the cell-cell adhesion complex in association with other proteins including catenin molecules (α-, β-, and γ) and p120 [21]. Loss of membranous localization of e-cadherin has been associated with invasion, metastasis and aggressive behavior in many human malignancies [21,22]. Loss of membranous staining with abnormal nuclear localization has been reported in pituitary, pancreatic, esophageal and urothelial tumors [21-24]. The mechanism of this abnormal localization is still unclear. Loss of membranous e-cadherin expression in urothelial carcinoma has been correlated with aggressive disease, increased risk of nodal metastasis and death [22]. Thomas et al. reported loss of membranous e-cadherin expression in approximately one-third of foci of PBA demonstrating signet-ring cell morphology in comparison to colonic type PBA; however, they did not encounter abnormal nuclear localization of e-cadherin [2].

In the current study, none of the CA or UC controls demonstrated nuclear staining pattern. E-cadherin molecule is comprised of 3 domains – extracellular, transmembrane and intracytoplasmic. Amongst the commercially available antibody clones, 36/E clone is directed against the cytoplasmic portion of e-cadherin. Our results of e-cadherin are based on use of this clone. (Table 1) The same clone was also used by the prior studies, which reported aberrant nuclear localization of e-cadherin [21-24].

CDX-2 is a mammalian homeobox gene, encoding a nuclear transcription factor, which is implicated as a tumor suppressor [16]. Nuclear staining is seen in normal colonic epithelial cells and colonic adenocarcinoma has been reported [3,25]. CDX-2 expression was seen in 83.3% cases of PBA, all cases of MCA and CA which is in accordance with the prior studies [3,14-16,25]. Interestingly, Suh et al. [16] also reported absence of CDX-2 expression in half of the cases of PBA. Two cases (16.7%) of PBA in our series were negative for CDX-2.

Villin is a 93-kilodalton actin-binding protein involved in the maintenance of brush border apparatus. Its expression can be seen in gastrointestinal tract and renal epithelial cells as well as in adenocarcinomas of gastrointestinal tract, pancreas, endometrium and ovary [16]. A limited number of studies have analyzed the role of villin in distinguishing PBA from SBA which reported overlapping staining between PBA and MCA [16-18]. We too did not find any difference in staining pattern between the two tumor groups.

The CK7 and CK20 staining profile has been used previously in distinguishing tumors from different sites. Gastrointestinal tumors, including colonic and rectal adenocarcinomas, tend to be CK20+ and CK7-. For urothelial neoplasms, the staining profile is the exact opposite. This difference was studied previously in PBA, but with limited success. Many PBA have been reported to show a CK20+ and CK7- profile, similar to colonic adenocarcinomas [3,15,17]. CK7 immunoexpression was seen in all cases of urothelial carcinoma and one third of PBAs. In contrast, only one (8.33%) case of SBA was positive for CK7 and all CAs were negative. CK20 immunostaining was seen in all PBAs, SBAs and colonic adenocarcinoma. Based on these findings, CK7 appeared to be of limited utility in distinguishing PBAs from secondary bladder adenocarcinomas and CK20 was not able to distinguish between the two tumor groups.

We also attempted to study the molecular aspect of PBA and MCA with a limited number of available cases (5 PBA and 4 MCA). FISH analysis using UroVysion probe sets were performed. To the best of our knowledge, our study is the first one that utilized molecular methods to attempt to distinguish PBA from MCA. In our series of nine cases, the predominant abnormality was homozygous loss of 9p21 followed by polysomy pattern and single chromosome gain (chromosomes 3, 7). These findings however did not appear to be helpful in distinguishing PBA from SBA. Kipp et al [26]. studied FISH findings using UroVysion probe sets in bladder carcinoma variants. In their study, the most frequent abnormality documented in bladder adenocarcinoma was 9p21 homozygous loss followed by single gain only. They did not find significant difference in the chromosomal abnormalities between the different tumor types. This study, unlike ours, did not compare bladder adenocarcinoma with metastatic adenocarcinoma.

## Conclusion

In summary, PBA is a rare and aggressive malignant neoplasm of the urinary bladder that morphologically mimics metastatic colorectal adenocarcinoma at the morphological level and to some extent immunohistochemically. A panel of antibodies comprised of β-catenin, e-cadherin, CK7 and CDX-2 can be helpful in distinguishing PBA from MCA in the light of appropriate clinical findings. Although FISH using the UroVysion probe set demonstrated various chromosomal abnormalities, it did not show significant differences between PBA and MCA. The results of the study reemphasize the persistence of the diagnostic challenge in distinguishing PBA and MCA clinico-pathologically. However, small cohort size was an important limitation of this study and therefore larger studies are required to substantiate the above conclusion. Moving further with question, It may be of interest to investigate the molecular landscape of the two tumors using high-throughput molecular analysis methods for possible diagnostic biomarkers.

## Competing interests

The authors declare that they have no competing interests.

## Authors’ contribution

MBA performed immunohistochemistry on all the cases in this study. KMC performed FISH studies on all these cases. MAS helped in case organization and critical manuscript review. SR wrote the manuscript, reviewed immunohistochemistry and FISH results and analyzed the data, AVP reviewed the immunohistochemistry and FISH results, critically reviewed the paper, arranged resources for the project and uploaded the manuscripts. All authors read and approved the final manuscript.
